# Editorial: AI in Biological and Biomedical Imaging

**DOI:** 10.3389/fmolb.2021.804476

**Published:** 2021-11-24

**Authors:** Xin Gao, Lihua Li, Min Xu

**Affiliations:** ^1^ King Abdullah University of Science and Technology (KAUST), Thuwal, Saudi Arabia; ^2^ Hangzhou Dianzi University, Hangzhou, China; ^3^ Carnegie Mellon University, Pittsburgh, PA, United States

**Keywords:** biomedical imaging, bioinformatics, AI, biological imaging, machine learning

Imaging is the visual representation of structures and functions of objects, such as biological molecules, biological ultrastructures, tissues, and the spatial organizations of the objects. It is also an indispensable step towards diagnostics and therapeutics in modern medicine. For example, during the current pandemic caused by COVID-19, CT-scans have been used, in addition to nucleic acid detection, as a main criterion for diagnostics. Unlike computers, the human brain has a remarkably strong ability to understand and interpret the information obtained from imaging data, more so than from interpreting numerical or textual data. On the other hand, AI methods may produce more objective and highly reproducible analysis results with increased automation. Therefore, it is beneficial to develop AI methods to complement manual image analysis.

Imaging is playing an increasingly significant role in both biological and biomedical sciences. With technologies including optical microscopy, fluorescence microscopy, electron tomography, nuclear magnetic resonance, single particle cryo-EM, and X-ray crystallography, biological imaging has provided rich information about biological systems and molecules at various resolutions, all the way from tissue-level, to cellular-level, to organelle-level, to macromolecular-level, to small-molecular-level, and to atomic-level. Imaging also has many diagnostic and therapeutic applications in medicine with different modalities, such as ultrasound, computed tomography (CT), magnetic resonance imaging (MRI), positron emission tomography (PET), and optical coherence tomography (OCT). Such technologies can provide fast, non-invasive, painless and direct information to clinicians and physicians, which is critical to not only diagnosis, but also prognosis and treatment.

With the recent development of AI technologies, especially deep learning, the frontiers on biological and biomedical imaging have been greatly advanced. In this Research Topic, we have collected 16 high quality works on developing or applying state-of-the-art AI techniques for processing, information mining, integrating, diagnosing, comparing, and reviewing biological and biomedical imaging, with their applications in biology, diagnostics and therapeutics.

There are in total 16 papers accepted by this Research Topic. Each paper was handled by one guest editor and reviewed by at least two reviewers. We are very grateful to the reviewers in helping us select these high-quality papers for this Research Topic.

The accepted papers focus on developing AI algorithms and systems to process, analyze, interpret, and mine from both biomedical and biological imaging data. We begin by providing a thought-provoking perspective on the future of radiology diagnostic service (Seong et al.). Radiology has been a leading technology of digital transformation in healthcare, which is again at the crossroad for the next generation of transformation, possibly evolving as a one-stop integrated diagnostic service. AI promises to offer radiology new powerful new digital tools to facilitate the next transformation. This paper proposes three pathways for AI’s role in radiology: (1) improving the performance of CAD, (2) improving the productivity of radiology service by AI-assisted workflow, and (3) developing radiomics that integrate the data from radiology, pathology, and genomics to facilitate the emergence of a new integrated diagnostic service.

We then present papers on 2D biomedical imaging data. We first discuss three papers on pathology imaging. Xu et al. proposed an effective immunohistochemistry pathology microscopic image-generation method that can generate synthetic immunohistochemistry pathology microscopic images from hematoxylin-eosin stained pathology microscopy images without any annotation. CycleGAN is adopted as the basic architecture for the unpaired and unannotated dataset. Moreover, multiple instances learning algorithms and the idea behind conditional GAN are considered to improve performance.

Liu et al. selected Ki-67-expression as the representative of molecular information. They proposed a method that can predict Ki-67 positive cells directly from H&E stained slides by a deep convolutional network model. To train this model, they constructed a dataset containing Ki-67 negative or positive cell images and background images. These images were all extracted from H&E stained WSIs and the Ki-67 expression was acquired from the corresponding IHC stained WSIs. The trained model was evaluated both on classification performance and the ability to quantify Ki-67 expression in H&E stained images.

He et al. proposed a hybrid-attention nested UNet (Han-Net), which consists of two modules: a hybrid nested U-shaped network (H-part) and a hybrid attention block (A-part). H-part combines a nested multi-depth U-shaped network and a dense network with full resolution to capture more effective features. A-part is used to explore attention information and build correlations between different pixels. With these two modules, Han-Net extracts discriminative features, which effectively segment the boundaries of not only complex and diverse nuclei but also small and dense nuclei. The comparison in a publicly available multi-organ dataset shows that the proposed model achieves the state-of-the-art performance compared to other models.

We further accepted two papers on X-ray data analysis. Yang et al. proposed a data-driven diagnostic model for hip dysplasia. Angles including CE, sharp, and Tonnis angle which are commonly measured in clinical diagnosis, are automatically obtained. Samples, each of which consists of these three angle values, are used for clustering according to their densities in a descending order. A three-dimensional normal distribution derived from the cluster is built and regarded as the parametric model for diagnosis of hip dysplasia.

Li et al. proposed an interpretable method called Deetal-Perio to predict the severity degree of periodontitis in dental panoramic radiographs. In their method, alveolar bone loss (ABL), the clinical hallmark for periodontitis diagnosis, could be interpreted as the key feature. To calculate ABL, they also proposed a method for teeth numbering and segmentation. First, Deetal-Perio segments and indexes the individual tooth via Mask R-CNN combined with a novel calibration method. Next, Deetal-Perio segments the contour of the alveolar bone and calculates a ratio for individual tooth to represent ABL. Finally, Deetal-Perio predicts the severity degree of periodontitis given the ratios of all the teeth.

We also accepted one paper on quantifying vascular density in tissue engineered constructs. Strobel et al. developed a semi-automated method that leverages machine learning to identify and quantify vascular metrics in an angiogenesis model imaged with different modalities. Their software, BioSegment, is designed to make high throughput vascular density measurements of fluorescent or phase contrast images.

We then present papers on 3D biomedical imaging data. We start from two papers on CT data analysis. Dou et al. aimed to test whether chest CT manifestation of 2019 novel coronavirus (COVID-19) can be differentiated by a radiologist or a computer-based CT image analysis system. They conducted a retrospective case-control study that included 52 laboratory-confirmed COVID-19 patients and 80 non-COVID-19 viral pneumonia patients. Their results do not support CT findings replacing microbiological diagnosis as a critical criterion for COVID-19 diagnosis.

Cai et al. developed and validated a radiomics-based nomogram to predict the prognosis of colorectal cancer (CRC). A total of 381 patients with colorectal cancer were enrolled and radiomic features were extracted from the vein phase of preoperative computed tomography. Their results show that radiomics score derived from the preoperative CT image was an independent prognostic factor and could be a complement to the current staging strategies of colorectal cancer.

We further accepted four papers on MRI data analysis. Fan et al. proposed a framework of a 3D-Mask region-based convolutional neural network (3D-Mask RCNN) computer-aided diagnosis (CAD) system for mass detection and segmentation with a comparative analysis of performance on patient subgroups with different clinicopathological characteristics. The results suggest that the 3D-Mask RCNN CAD framework has advantages over 2D-based mass detection on both the whole data and subgroups with different characteristics.

Fan et al. predicted responses to NACT in breast cancer by analyzing early changes in tumor heterogeneity modeled by longitudinal dynamic contrast-enhanced magnetic resonance imaging (DCE-MRI). Their results suggested that changes in DCE-MRI features that reflect a reduction in tumor heterogeneity following NACT could provide early prediction of breast tumor response.

Wang et al. proposed a novel model structure to capture 3D MRI images’ essential information and converted them into lower dimensions. The novel CNN model they proposed could automatically differentiate the rare NMOSD from MS, especially, our model showed better performance than traditional 3D CNN models.

Gu et al. proposed a multi-head self-attention model (MSAM). By integrating the self-attention mechanism and multilayer perceptron method, the MSAM offers a promising tool to enhance the classification of Temporal Lobe Epilepsy (TLE) subtypes. The robustness of MSAM is extensively assessed with various ablation tests, which demonstrates the effectiveness and generalizability of the proposed approach.

We then included a paper on developing diagnostic algorithm for capsule endoscopy, which is a leading diagnostic tool for small bowel lesions. Kong et al. proposed a multi-task framework, called the multi-task classification and segmentation network (MTCSN), to achieve joint learning of clearness degree and tissue semantic segmentation. Extensive experiments and ablation studies report the significant performance gains of the MTCSN over state-of-the-art methods.

We further accepted a paper on reviewing tactile perception technologies on minimally invasive surgery (MIS), which has been the preferred surgery approach owing to its advantages over conventional open surgery. As a major limitation, the lack of tactile perception impairs the ability of surgeons in tissue distinction and maneuvers. Huang et al. aimed to provide potential tactile perception methods for MIS by reviewing literatures on tactile sensing in MIS and literatures on industrial robotic tactile perception technologies, especially AI methods on tactile images.

We finish our collection of papers by including one paper on biological imaging. Zhou et al. developed a one-shot learning framework, called cryo-ET one-shot network (COS-Net), for simultaneous classification of macromolecular structure and generation of the voxel-level 3D segmentation, using only one training sample per class, from cryo-electron tomography data. Their experimental results demonstrated that COS-Net could efficiently classify macromolecular structures with small amounts of samples and produce accurate 3D segmentation at the same time.

To conclude, we thank the authors and the reviewers for their contribution to this Research Topic. We are confident that the collection of articles in this Research Topic will serve as an inspiring compendium for future AI advancement and deployment in biomedical and biological imaging fields.

